# Susceptibility for Lupus Nephritis by Low Copy Number of the *FCGR3B* Gene Is Linked to Increased Levels of Pathogenic Autoantibodies

**DOI:** 10.1155/2013/750814

**Published:** 2013-06-20

**Authors:** Johannes C. Nossent, Andrea Becker-Merok, Maureen Rischmueller, Sue Lester

**Affiliations:** ^1^Department of Rheumatology, Institute of Clinical Medicine, University of Tromsø, 9037 Tromsø, Norway; ^2^Department of Rheumatology, University Hospital Northern Norway, P.O. Box 14, 9038 Tromsø, Norway; ^3^Department of Rheumatology, Basil Hetzel Institute, The Queen Elizabeth Hospital, Adelaide, SA 5020, Australia; ^4^Division of Medicine, University of Adelaide, Adelaide, SA 5000, Australia

## Abstract

Low copy number (CN) of the *FCGR3B* gene reduces *FCGR3B* membrane expression on neutrophils and results in clearance of a smaller amount of immune complex. We investigated *FCGR3B* CN in relation to the clinical phenotype in a Caucasian SLE cohort (*n* = 107). *FCGR3B* CN was determined by three different qPCR parameter estimations (Ct−, Cy0, and cpD1) and confirmed by the FCGR2C/FCGR2A paralog ratio test. Clinical and serological data were then analyzed for their association with *FCGR3B* CN. Low *FCGR3B* CN (<2) was more frequent in SLE patients than in healthy controls (*n* = 162) (20% versus 6%, OR 4.15, *P* = 0.003) and associated with higher disease activity scores (SLEDAI 10.4 versus 6.1, *P* = 0.03), lupus nephritis (LN) (25 versus 5%, *P* = 0.03), and increased levels of antibodies against dsDNA (81 versus 37 IU, *P* = 0.03), C1q (22 versus 6 IU, *P* = 0.003), and ribosomal P (10 versus 5 IU, *P* = 0.01). No such associations were seen with antibodies against extractable nuclear antigens or high *FCGR3B* CN (>2). In multivariate analyses, LN was independently associated with anti-C1q-Ab levels (*P* = 0.03) and low *FCGR3B* CN (*P* = 0.09). We conclude that the susceptibility for LN in patients with low *FCGR3B* CN is linked to increased levels of pathogenic autoantibodies.

## 1. Introduction

Systemic lupus erythematosus (SLE) is a severe autoimmune disease that causes a wide spectrum of clinical and serological abnormalities [1]. The characteristic production of antibodies against nuclear and cytoplasmic antigens, which normally are shielded from the immune system, results among others from defective clearance of apoptotic material [2, 3]. Immune complexes containing autoantibodies and nuclear antigens (IC) play a central role in renal inflammation, and depending on their interaction with activating and/or inhibitory FCgR can lead to complement activation, cytokine release, and attraction of neutrophils in Lupus Nephritis (LN), similar to findings in anti-GBM disease [4–6]. Although an abundance of autoantibodies can be detected in sera from SLE patients, only a limited number of autoantibodies are associated with specific disease manifestations such as anti-dsDNA and LN [1]. In contrast, antibodies against other intracellular antigens, such as anti-Ro/La, are in general consistently present in stable titers over prolonged periods of time, independent of underlying disease activity [7]. 

Copy number variation (CNV) designates the presence of duplications or deletions of DNA segments of considerable size (more than 1 kb) and may be present in as much of 12% of the human genome [8, 9]. CNV is increasingly recognized as an important genetic predisposing factor for complex diseases including SLE and RA [10, 11]. *FCGR3B* is an activating membrane glycoprotein expressed specifically by human neutrophils (and stimulated eosinophils) and preferentially interacts with complexed IgG [12]. IgG binding initiates a neutrophil effector response resulting in the clearance of IC [13, 14]. The *FCGR3B* gene is carried in the FCgR cluster on chromosome 1 (1q23), and low *FCGR3B* CN has emerged as a susceptibility factor for SLE in case-controls studies with different ethnic backgrounds [15–18]. There is however limited information on the relationship between low *FCGR3B* CN and clinical phenotypes in SLE. An association between low *FCGR3B* CN and LN has been observed in Caucasian patients as well as in experimental lupus, but several studies have been unable to replicate these associations in Asian or Afro-American patients [16, 18, 19]. Despite the role of *FCGR3B* in clearance of immune complexes, none of these studies have been able to demonstrate the presumed link between low *FCGR3B* CN and pathogenic IgG autoantibodies. Given the limited data available, we therefore investigated the potential association between *FCGR3B* CNV and relevant disease manifestations in SLE. 

## 2. Patients and Methods

The study included 107 Caucasian SLE patients (87% female, median age 47 years), all fulfilling 1997 ACR criteria for SLE classification and 162 population-based Caucasian healthy controls (53% females, median age 56 years). Patient selection, disease activity (SLEDAI-2K), and accrued organ damage assessment (SLICC-DI) have been detailed before [20] (see Supplementary Tables 1 and 2 in the Supplementary Material available online at http://dx.doi.org/10.1155/2013/750814). The study was conducted in accordance with the Declaration of Helsinki and approved by the Ethics Committees, and all participants gave written informed consent. 

### 2.1. CN Quantification for *FCGR3B* Gene

Genomic DNA was extracted from frozen PBMCs using DNeasy Mini Kit (Qiagen, Hilden, Germany), following the manufacturer's instructions. Samples were quantitated using a Nanodrop ND-1000 Spectrophotometer (NanoDrop Technologies). CN determination was performed using 20 ng genomic DNA in an ABI 7300 real-time PCR system using a custom TaqMan copy number assay (Applied Biosystems, Hs04211858-cn). Briefly, the *FCGR3B* primers specifically amplified *FCGR3B* within intron 3, and fluorescence detection utilized a dual-labeled FAM-MGB probe. TaqMan copy number RNase P (Applied Biosystems, product 4403326), with a dual-labeled VIC-TAMRA probe, was used as the reference (2CN) assay. Duplex PCR reactions were performed in triplicate with fluorescence signals normalized to ROX. Each test run included three reference samples (1, 2, and 3 *FCGR3B* copies) to control for potential batch variation. 

*FCGR3B* copy number was analyzed using Copy Caller software (v.1.0, Applied Biosystems, USA); results were accepted only when the calling confidence for discrete CN assignment was >80%, and the dCq standard deviation between replicates was <0.20; otherwise samples were retested. CN assignment was compared for three different quantitation points (Cq): (i) Ct, with auto-baselining and 0, 2 threshold, as recommended by the manufacturer; (ii) Cy0, which is less sensitive to amplification inhibitors [2], again estimated using autobaselined data; (iii) cpD1 (second derivative maximum) estimated from unbaselined data (determined from a 6-parameter log-logistic curve which explicitly models the baseline). The Cy0 and cpD1 estimates were determined using the R qpcR library [2, 21, 22]. The 3 quantitation points gave equivalent CN assignments. However, occasional, sporadic FAM autobaselining problems were observed, which if undetected, resulted in spuriously high CN assignment. Repeat testing confirmed that the cpD1 estimate from unbaselined data was therefore the most reliable.

The *FCGR3B* qPCR CNV assay was validated against the *FCGR2C*/*FCGR2A* paralog ratio, end point, and PCR assay [15]. As *FCGR2C* CN is in complete linkage with *FCGR3B* CN and *FCGR2A* does not exhibit CNV [15, 23], *FCGR2C*/*FCGR2A* ratio thus offers independent confirmation of *FCGR3B* CN [15, 23]. Briefly, a single primer pair was used to coamplify FCGR2C and FCGR2A, and the amplicons corresponding to each gene (274 and 279 bp, resp.) were quantified following capillary electrophoresis (QIAxcel, USA). One hundred samples were tested in parallel for both assays. The methodology has been described in detail before [24]. There was complete concordance between the independently assigned CN for the *FCGR3B* qPCR assay and the *FCGR2C* paralog ratio end-point PCR assay for 99 samples; one sample was not assigned by the paralog ratio method and was not retested. 

### 2.2. Serological Assays

IgG class antibodies against double-stranded DNA (anti-dsDNA) and Ro/La were routinely tested in our immunology laboratory by enzyme immunoassays (Elia, Phadia, Freiburg, Germany), while antibody levels against nucleosomes, C1q, and ribosomal protein P were determined by ELISA (Orgentec Diagnostika, Mainz, Germany) and BAFF by a sandwich enzyme immunoassay (Quantikine Immunoassay; R&D Systems, Minneapolis, MN, USA) on stored serum aliquots (−20°C). All assays performed in duplex following the manufacturers' recommendations. 

### 2.3. Statistics

Numbers represent medians unless specified otherwise. The association between *FCGR3B* CN and disease susceptibility was analyzed by odds ratios (OR). Comparisons of median levels between groups were done by Mann-Whitney *U* test, while Spearman's correlation coefficient of ranked values was used to determine the strength of relations between continuous variables and *χ* ^2^ test to test associations between discontinuous variables. Resulting *P*-values <0.05 were considered indicative of statistical significance. Clinical and serological disease features associated with LN by logistic regression analyses were subsequently entered into multivariate backward modeling based on Wald statistics (*P* < 0.1 to enter) to determine their independent relationship with LN. 

## 3. Results

The *FCGR3B* CN distribution (Table 1) was significantly different in SLE patients compared to controls (*χ* ^2^ 13.8, *P* = 0.001). When compared with the normal diploid 2 CN, low *FCGR3B* CN (≤1) was increased in frequency in SLE (OR 4.15, 95% CI: 1.82–9.46, *P* = 0.0003), while there was no association with high *FCGR3B *CN (≥3, *P* = 0.82) (Table 1).

Within the SLE cohort, low *FCGR3B* CN was associated with higher SLEDAI-2K scores (*P* = 0.03, Table 2). Low *FCGR3B* CN was also increased in frequency in patients with active renal disease (characterized by proteinuria with active urinary sediment) (25 versus 6.4%, *P* = 0.03) (Table 2). Low *FCGR3B* CN was not associated with onset age or gender, arthritis, skin disease or other manifestation of active disease as defined in SLEDAI-2 K, total or organ system-specific scores SDI scores.

In terms of serological/laboratory findings, low *FCGR3B *CN was associated with a higher prevalence and increased levels of anti-dsDNA, anti-C1q, and antiribosomal P autoantibodies (Table 2). *FCGR3B *CN was however unrelated to anti-Ro prevalence, RF titers, ESR, hemoglobin leukocyte or lymphocyte counts, C3, and s-BAFF levels (data not shown). In nonparametric correlation analyses, *FCGR3B* CN was inversely and significantly correlated with anti-dsDNA (Rs −0.24; *P* = 0.015), antiribosomal P (Rs −0.31; *P* = 0.002), and anti-C1q (Rs −0.251; *P* = 0.01) autoantibody titers, but not to RF (Rs 0.12; *P* > 0.3). Upon multivariate logistic regression analysis, the final model confirmed an independent relation with LN for anti-C1q antibody levels (OR 1.03, *P* = 0.026), while the relation with low *FCGR3B* CN reached only borderline statistical significance despite its large effect size effect (OR 4.2, *P* = 0.09) (Table 3). 

## 4. Discussion

*FCGR3B* is a stimulatory receptor located predominantly on the cell surface of neutrophils and belongs to the family of Fc gamma receptors, which are primarily involved in IC clearance [13]. This study confirms that low *FCGR3B* CN is a susceptibility factor for SLE [13, 14, 17–19]. In addition, we show for the first time a link between low *FCGR3B* CN and the presence and titers of specific pathogenic autoantibodies in SLE. 

*FCGR3B* CN variation has become established as a risk factor for a range of systemic autoimmune diseases, including RA, Sjögren's syndrome, and SLE [11]. This reflects a growing realization that CNV is frequent throughout the genome and may be important genetic risk factors for complex disease [9, 25, 26]. The observed effect size for this association (OR 4.2) was larger than in previously reported studies but falls within the confidence interval reported in a recent meta-analysis [11]. 

While the association between low *FCGR3B *CN and SLE susceptibility is firmly established, there is still limited knowledge about the mechanism by which *FCGR3B* CN contributes to this disease [1, 27]. Our data support an association between low *FCGR3B* CN and the prevalence of LN in Caucasian patients, while no such association has been observed in in Chinese and Hispanic patients [16, 19, 28]. Other studies have demonstrated that single nucleotide polymorphisms (SNPs), but not CNV of the *FCGR3B* gene, constituted a risk factor for LN in Chinese and other SLE cohorts [12, 25, 28]. Morris et al. have shown that there is a combined effect of *FCGR3B* CNV and the presence of NA1/NA2 SNP on disease susceptibility [29], and it is clearly of interest to further investigate how combined changes in *FCGR3B* gene copy number and product structure impact on disease susceptibility and clinical phenotypes.

Low *FCGR3B* CN was not associated with any other clinical manifestation of SLE in this study and other studies [16, 19, 28]. The association with higher SLEDAI scores is likely a reflection of the higher scores obtained by patients with LN. 

The biological role of *FCGR3B* is not fully elucidated. *FCGR3B* lacks a transmembrane domain and must interact with other membrane receptors such as FCGR2A and β2-integrin (CD11/CD18b) to fully activate neutrophils [30–32]. In vitro cross-linking of *FCGR3B* induces calcium mobilization and tyrosine phosphorylation and leads to tethering of neutrophils to immobilized ICs and IgG phagocytosis [31, 33, 34]. In healthy controls, low *FCGR3B *CN reduces the adherence of neutrophils to IC and subsequent IC clearance [35], and it seems reasonable to assume a similar effect in SLE patients. The anticipated consequence would be higher levels of circulating IC/autoantibodies, but this has not been documented in human SLE. This study is one of the first to demonstrate a link between low *FCGR3B* CN and high titers of antibodies against dsDNA, C1q, and ribosomal protein P in SLE patients. These autoantibodies are among the most disease-specific autoantibodies in SLE, and in particular, anti-dsDNA and anti-C1q are implicated in the pathogenesis of LN [1, 36]. Our data suggest that these circulating autoantibodies represent a potential connection between low *FCGR3B* CN and human lupus, especially LN [19, 37]. 

Antibodies against Ro/La, Sm, and IgG (RF) were not associated with variation in *FCGR3B* CN in this SLE cohort. Compared to anti-dsDNA and anti-C1q autoantibodies, both RF and anti-Ro/La are less disease specific and do not fluctuate with disease activity [1]. RF exerts a differential effect on IC clearance, and depending on characteristics such as antibody excess and glycosylation of the Fc fragment may increase or decrease IC uptake [32, 38]. Data from RA studies have also failed to show a convincing association between low *FCGR3B* CN and RF or antibodies against citrullinated peptides (ACPA) [24, 39], while studies in Sjögren's syndrome patients failed to find an association with anti-Ro antibodies [40, 41]. An association between renal disease and anti-Sm has been described in African-American SLE patients who tend to have a fourfold higher prevalence of anti-Sm than Caucasian patients [42]. The distribution of anti-Sm was similar between patients with low or high *FCGR3B* CN as was otherwise the case for other antibodies against ENA, which all have a less distinctive association with clinical disease. While a potential role for *FCGR3B* CNV and anti-Sm could not be substantiated in this Caucasian cohort, this does not exclude a potential association in other cohorts. Together, these data indicate that the specificity of an antibody is of major importance in the interaction with *FCGR3B*, and more detailed study of the functional consequences of *FCGR3B* stimulation by SLE-specific (complexed as well as monomeric) autoantibodies will be highly informative.

The strength of this study lies in the integrated approach for measuring CN variation that included extensive data quality checking as well as independent validation by a separate assay. This methodology resulted in high confidence and discrete CN calls which have emerged as important aspect of *FCGR3B* CNV determination [11, 43]. Nonetheless, every method for CNV study presents its own unique challenges making direct comparisons difficult, especially given with regards to the newer microarray-based technologies [25].

Another asset of this study is the uniformity of data ensured by simultaneous collection of clinical and laboratory data in a genetically homogenous cohort from a single centre and subsequent uniform ELISA-based testing of expanded autoantibody profiles. The main limitation of this study is the moderate cohort size. While a larger sample size would have increased the power of our findings, it would not be anticipated to effectively change the results as sample size was not widely different from other studies where the impact of *FCGR3B* CNV on disease susceptibility was described [24, 41]. Also, serological data on circulating immune complexes (CIC) were not available as CIC measurement is no longer routinely practiced. CIC assays do not provide insight into the specificity of the antigen-antibody interaction [44, 45]. Particularly important, CIC assays do not differentiate antigen-complexed antibody from non-specifically aggregated or monomeric autoantibody asC1qBA results are confounded by monomeric immunoglobulin such as anti-C1q in serum of SLE patients [46]. 

## 5. Conclusions 

We confirm that low *FCGR3B* CN is a risk factor for LN in Caucasian SLE patients, likely through a selective reduction in the clearance of disease-specific autoantibodies only. This suggests that *FCGR3B* is involved in removal of a limited repertoire of pathogenic autoantibodies in SLE. 

## Supplementary Material

Supplemental Table 1: Overview of the assessment of the SLE disease activity index (SLEDAI) used in this study showing the absolute scores and definitions for their use. The maximum score is 104.
Supplemental Table 2: Provides the details for the assessment of the SLICC damage index (SDI) used in this study. Scores are only registered if a specific type of organ damage has been present for >6 months, irrespective of its cause.

## Figures and Tables

**Table 1:** Frequency of copy number (CN) variation in the *FCGR3B* gene in SLE patients and controls. Odds ratios with 95% confidence intervals (CI) for the association between *FCGR3B* gene copy number (CN) and SLE. Reference group is healthy controls (*n* = 162).

*FCGR3B * CN	SLE *n* = 107	Controls *n* = 162	OR	*P* value
<2	20%	6%	4.15 (CI: 1.82–29.46)	*P* = 0.0003
2	70%	86%	1	
>2	9%	8%	1.03 (CI: 0.43–2.47)	*P* = 0.82

Global *χ* ^2^ = 13.78, degrees of freedom = 2, and *P* = 0.001.

**Table 2:** Univariate analysis comparing clinical features between SLE patients with low (<2) *FCGR3B* and ≥2 *FCGR3B* gene copy number. Figures indicate mean values or number of patients (percentage). *P* values derive from Mann-Whitney *U* test and chi-square testing as appropriate.

	*FCGR3B* < 2 (*n* = 21)	*FCGR3B* ≥ 2 (*n* = 86)	*P* value
Age (yrs)	43.3	47.9	0.3
Female gender (%)	18 (86)	77 (90)	0.47
Disease duration (months)	162	151	0.7
Organ damage present (SDI > 0)	60	58	0.9
SLEDAI-2K score	10.4	6.1	0.03
LN present (%)	5 (24)	6 (6.9)	0.03
Arthritis present (%)	4 (19)	9 (11)	0.3
Active skin lesions present (%)	2 (10)	3 (4)	0.4
Leukocytopenia (<4)	3 (14)	18 (21)	0.3
Anti-dsDNA positive	8 (38)	16 (18)	0.05
Anti-dsDNA titer (*n* < 55 IU)	81	37	0.03
Anti-C1q positive	6 (29)	10 (12)	0.06
Anti-C1q titer (*n* < 11 IU)	22	5.5	0.003
Antiribosomal P positive	4 (19)	6 (7)	0.1
Antiribosomal P titer (*n* < 8 IU)	10	5	0.01
Anti-Ro positive (%)	43	36	0.62
Anti-Sm positive (%)	5	6	0.9
Low C3 (<81 mg/dL) (%)	30	28	0.9

SLEDAI-2K: SLE Disease Activity Index-2000 version, SDI: SLICC-ACR Damage Index, LN: lupus nephritis (see methods for definition), and C3: complement factor 3.

**Table 3:** Odds ratios (OR) for the association between disease characteristics and presence of LN. OR correspond to Exp (β) in logistic regression analyses with presence of active LN as the dependent parameter (see methods for detailed description). “—” indicates nonsignificant findings upon multivariate analysis.

	Univariate			Multivariate		
	Exp (β)	CI	*P* value	Exp (β)	CI	*P* value
*FCGR3B* CN < 2	4.8	1.25–18.9	0.02	4.2	0.8–23.6	0.09
Serum albumin	0.87	0.77–0.99	0.047	—	—	—
Anti-dsDNA Ab	1.01	1.003–1.018	0.008	—	—	—
Anti-C1q Ab	1.04	1.01–1.06	0.003	1.03	1.03–1.05	0.026
Low C3	7.1	1.6–29.8	0.007	—	—	—

## References

[1] Rahman A, Isenberg DA (2008). Systemic lupus erythematosus. *The New England Journal of Medicine*.

[2] Guescini M, Sisti D, Rocchi MBL, Stocchi L, Stocchi V (2008). A new real-time PCR method to overcome significant quantitative inaccuracy due to slight amplification inhibition. *BMC Bioinformatics*.

[3] Kontaki E, Boumpas DT (2010). Innate immunity in systemic lupus erythematosus: sensing endogenous nucleic acids. *Journal of Autoimmunity*.

[4] Lauterbach M, O’Donnell P, Asano K, Mayadas TN (2008). Role of TNF priming and adhesion molecules in neutrophil recruitment to intravascular immune complexes. *Journal of Leukocyte Biology*.

[5] Zhou X, Lv J, Yu L (2010). FCGR2B gene polymorphism rather than FCGR2A, FCGR3A and FCGR3B is associated with anti-GBM disease in Chinese. *Nephrology Dialysis Transplantation*.

[6] Zhou X, Lv J, Bu D (2009). Copy number variation of FCGR3A rather than FCGR3B and FCGR2B is associated with susceptibility to anti-GBM disease. *International Immunology*.

[7] Niewold TB, Rivera TL, Buyon JP, Crow MK (2008). Serum type I interferon activity is dependent on maternal diagnosis in anti-SSA/Ro-positive mothers of children with neonatal lupus. *Arthritis and Rheumatism*.

[8] Armour KL, Smith CS, Clark MR (2010). Expression of human Fc*γ*RIIIa as a GPI-linked molecule on CHO cells to enable measurement of human IgG binding. *Journal of Immunological Methods*.

[9] Choy KW, Setlur SR, Lee C, Lau TK (2010). The impact of human copy number variation on a new era of genetic testing. *BJOG*.

[10] Schrider DR, Hahn MW (2010). Gene copy-number polymorphism in nature. *Proceedings of the Royal Society B*.

[11] McKinney C, Merriman TR (2012). Meta-analysis confirms a role for deletion in FCGR3B in autoimmune phenotypes. *Human Molecular Genetics*.

[12] Mayadas TN, Tsokos GC, Tsuboi N (2009). Mechanisms of immune complex-mediated neutrophil recruitment and tissue injury. *Circulation*.

[13] Niederer HA, Clatworthy MR, Willcocks LC, Smith KG (2010). FcgammaRIIB, FcgammaRIIIB, and systemic lupus erythematosus. *Annals of the New York Academy of Sciences*.

[14] Li X, Ptacek TS, Brown EE, Edberg JC (2009). Fc*γ* receptors: structure, function and role as genetic risk factors in SLE. *Genes and Immunity*.

[15] Niederer HA, Willcocks LC, Rayner TF (2010). Copy number, linkage disequilibrium and disease association in the FCGR locus. *Human Molecular Genetics*.

[16] Lv J, Yang Y, Zhou X (2010). FCGR3B copy number variation is not associated with lupus nephritis in a Chinese population. *Lupus*.

[17] Fanciulli M, Norsworthy PJ, Petretto E (2007). FCGR3B copy number variation is associated with susceptibility to systemic, but not organ-specific, autoimmunity. *Nature Genetics*.

[18] Molokhia M, Fanciulli M, Petretto E (2011). FCGR3B copy number variation is associated with systemic lupus erythematosus risk in Afro-Caribbeans. *Rheumatology*.

[19] Aitman TJ, Dong R, Vyse TJ (2006). Copy number polymorphism in Fcgr3 predisposes to glomerulonephritis in rats and humans. *Nature*.

[20] Eilertsen GO, Becker-Merok A, Nossent JC (2009). The influence of the 1997 updated classification criteria for systemic lupus erythematosus: epidemiology, disease presentation, and patient management. *The Journal of Rheumatology*.

[21] R Development Core Team (2008). *R: A Language and Environment for Statistical Computing*.

[22] Ritz C, Spiess A (2008). qpcR: an R package for sigmoidal model selection in quantitative real-time polymerase chain reaction analysis. *Bioinformatics*.

[23] Breunis WB, van Mirre E, Geissler J (2009). Copy number variation at the FCGR locus includes FCGR3A, FCGR2C and FCGR3B but not FCGR2A and FCGR2B. *Human Mutation*.

[24] Graf SW, Lester S, Nossent JC (2012). Low copy number of the FCGR3B gene and rheumatoid arthritis: a case-control study and meta-analysis. *Arthritis Research and Therapy*.

[25] Wain LV, Tobin MD (2011). Copy number variation. *Methods in Molecular Biology*.

[26] Calus MPL, De Koning DJ, Haley CS (2010). Including copy number variation in association studies to predict genotypic values. *Genetics Research*.

[27] Lee C, Scherer SW (2010). The clinical context of copy number variation in the human genome. *Expert Reviews in Molecular Medicine*.

[28] Yang H, Zhou Q, Chen DJ, Jiang H, Mao YY, Chen JH (2010). The single nucleotide polymorphisms gene but not the copy number variation of Fcgr3B is associated with lupus nephritis in Chinese people. *Lupus*.

[29] Morris DL, Roberts AL, Witherden AS (2010). Evidence for both copy number and allelic (NA1/NA2) risk at the FCGR3B locus in systemic lupus erythematosus. *European Journal of Human Genetics*.

[30] Cullere X, Lauterbach M, Tsuboi N, Mayadas TN (2008). Neutrophil-selective CD18 silencing using RNA interference in vivo. *Blood*.

[31] Tsuboi N, Asano K, Lauterbach M, Mayadas TN (2008). Human neutrophil Fcgamma receptors initiate and play specialized nonredundant roles in antibody-mediated inflammatory diseases. *Immunity*.

[32] Hogben DN, Devey ME (1986). Studies on rheumatoid factor: i. The effect of rheumatoid factor on the clearance of preformed immune complexes in mice. *Clinical and Experimental Immunology*.

[33] Marois L, Paré G, Vaillancourt M, Rollet-Labelle E, Naccache PH (2011). Fc*γ*RIIIb triggers raft-dependent calcium influx in IgG-mediated responses in human neutrophils. *The Journal of Biological Chemistry*.

[34] St-Onge M, Lagarde S, Laflamme C (2010). Proteinase-activated receptor-2 up-regulation by Fc*γ*-receptor activation in human neutrophils. *The FASEB Journal*.

[35] Willcocks LC, Lyons PA, Clatworthy MR (2008). Copy number of FCGR3B, which is associated with systemic lupus erythematosus, correlates with protein expression and immune complex uptake. *Journal of Experimental Medicine*.

[36] Riboldi P, Gerosa M, Moroni G (2005). Anti-DNA antibodies: a diagnostic and prognostic tool for systemic lupus erythematosus?. *Autoimmunity*.

[37] Stokol T, O’Donnell P, Xiao L (2004). C1q governs deposition of circulating immune complexes and leukocyte Fc*γ* receptors mediate subsequent neutrophil recruitment. *Journal of Experimental Medicine*.

[38] Devey ME, Hogben DN (1989). Rheumatoid factor and immune complexes. *Monographs in Allergy*.

[39] McKinney C, Fanciulli M, Merriman ME (2010). Association of variation in Fc*γ* receptor 3B gene copy number with rheumatoid arthritis in Caucasian samples. *Annals of the Rheumatic Diseases*.

[40] Nossent JC, Rischmueller M, Lester S (2012). Low copy number of the Fc-Gamma receptor 3B gene FCGR3B is a risk factor for primary Sjogren's syndrome. *The Journal of Rheumatology*.

[41] Mamtani M, Anaya J-M, He W, Ahuja SK (2010). Association of copy number variation in the FCGR3B gene with risk of autoimmune diseases. *Genes and Immunity*.

[42] Janwityanuchit S, Verasertniyom O, Vanichapuntu M, Vatanasuk M (1993). Anti-Sm: its predictive value in systemic lupus erythematosus. *Clinical Rheumatology*.

[43] Hollox EJ, Detering J, Dehnugara T (2009). An integrated approach for measuring copy number variation at the FCGR3 (CD16) locus. *Human Mutation*.

[44] Lloyd W, Schur PH (1981). Immune complexes, complement, and anti-DNA in exacerbations of systemic lupus erythematosus (SLE). *Medicine*.

[45] Swaak AJG, Groenwold J, Hannema A, Hack CE (1985). Correlation of disease activity with circulating immune complexes (C(1Q)bA) and complement breakdown products (C(3D)) in patients with systemic lupus erythematosus. A prospective study. *Rheumatology International*.

[46] Agnello V (1983). Immune complex assays in rheumatic diseases. *Human Pathology*.

