# Supplementary material for: Susceptibility for Lupus Nephritis by Low Copy Number of the FCGR3B Gene Is Linked to Increased Levels of Pathogenic Autoantibodies
Source: Autoimmune Dis. 2013 Jun 20;2013:750814. doi: 10.1155/2013/750814 (PMC3705838; doi:10.1155/2013/750814)
Supplement: Supplementary file 1 — Supplemental Table 1: Overview of the assessment of the SLE disease activity index (SLEDAI) used in this study showing the absolute scores and definitions for their use. The maximum score is 104. Supplemental Table 2: Provides the details for the assessment of the SLICC damage index (SDI) used in this study. Scores are only registered if a specific type of organ damage has been present for >6 months, irrespective of its cause. [file 750814.f1.pdf]

S. Table 1 SLEDAI (data collection sheet)

SLEDAI

(Enter weight in SLEDAI. Score column if descriptor is present at the time of the visit or in the preceding 10 days)

Study No.: **Patient name:** **Visit date:** day month year

Weight	SCORE	Descriptor	Definition
8	________	Seizure	Recent onset, exclude metabolic, infectious, or drug causes.
8	________	Psychosis	Altered ability to function in normal activity due to severe disturbance in the perception of reality. Include hallucinations, incoherence, marked illogical thinking, bizarre, disorganized, or catatonic behaviour. Exclude uraemia and drug causes.
8	________	Organic brain syndrome	Altered mental function with impaired orientation, memory, or other intellectual function, with rapid onset and fluctuating clinical features, inability to sustain attention to environment, plus at least 2 of the following: perceptual disturbance, incoherent speech, insomnia or daytime drowsiness, or increase or decrease psychomotor activity. Exclude metabolic, infectious or drug causes.
8	________	Visual disturbance	Retinal changes of SLE. Include cytoid bodies, retinal hemorrhages, serous exudates or hemorrhages in the choroid, or optic neuritis. Exclude hypertension, infection, or drug causes
8	________	Cranial nerve disorder	New onset of sensory or motor neuropathy involving cranial nerves.
8	________	Lupus headache	Severe, persistent headache; may be migrainous, but must be nonresponsive to narcotic analgesia.
8	________	CVA	New onset of cerebrovascular accident(s). Exclude arteriosclerosis.
8	________	Vasculitis	Ulceration, gangrene, tender finger nodules, periungual infarction, splinter hemorrhages, or biopsy or angiogram proof of vasculitis.
4	________	Arthritis	$\geq$ 2 joints with pain and signs of inflammation (i.e., tenderness, swelling or effusion).
4	________	Myositis	Proximal muscle aching/weakness, associated with elevated creatine phosphokinase/aldolase or electromyogram changes or a biopsy showing myositis.
4	________	Urinary casts	Heme-granular or red blood cell casts.
4	________	Hematuria	> 5 red blood cells/high power field. Exclude stone, infection or other cause.
4	________	Proteinuria	>0.5 gram/24 hours
4	________	Pyuria	>5 with blood cells/high power field. Exclude infection.
2	________	Rash	Inflammatory type rash.
2	________	Alopecia	Abnormal, patchy or diffuse loss of hair.
2	________	Mucosal ulcers	Oral or nasal ulcerations.
2	________	Pleurisy	Pleuritic chest pain with pleural rub or effusion, or pleural thickening.
2	________	Pericarditis	Pericardial pain with at least 1 of the following: rub, effusion, or electrocardiogram or echocardiogram confirmation.
2	________	Low complement	Decrease in CH50, C3 or C4 below the lower limit of normal for testing laboratory.
2	________	Increased DNA binding	Increased DNA binding by Farr assay above normal range for testing laboratory.
1	________	Fever	>38° C. Exclude infectious cause.
1	________	Thrombocytopenia	<100,000 platelets / $x10^9$ /L, exclude drug causes.
1	________	Leukopenia	<3,000 white blood cells / $x10^9$ /L, exclude drug causes.

TOTAL SCORE ________

S. Table 2 The SLICC/ACR damage index for SLE

Item	Score
Ocular (either eye, by clinical assessment)	
Any cataract ever	1
Retinal change *or* optic atrophy	1
Neuropsychiatric	
Cognitive impairment (e.g., memory deficit, difficulty with calculation, poor concentration, difficulty in spoken or written language, impaired performance level) *or* major psychosis	1
Seizures requiring therapy for 6 months	1
Cerebrovascular accident ever (score 2 if > 1)	1 (2)
Cranial or peripheral neuropathy (excluding optic)	1
Transverse myelitis	1
Renal	
Estimated or measured glomerular filtration rate <50%	1
Proteinuria $\geq$ 3.5 mg/24 hours	1
or	
End-stage renal disease (regardless of dialysis or transplantation)	3
Pulmonary	
Pulmonary hypertension (right ventricular prominence, or loud P2)	1
Pulmonary fibrosis (physical and radiograph)	1
Shrinking lung (radiograph)	1
Pleural fibrosis (radiograph)	1
Pulmonary infarction (radiograph)	1
Cardiovascular	
Angina *or* coronary artery bypass	1
Myocardial infarction ever (score 2 if > 1)	1 (2)
Cardiomyopathy (ventricular dysfunction)	1
Valvular disease (diastolic murmur, or systolic > 3/6)	1
Pericarditis for 6 month, *or* pericardiectomi	1
Peripheral vascular	
Claudicatio for 6 months	1
Minor tissue loss (pulp space)	1
Significant tissue loss ever (e.g., loss of digit or limb) (score 2 if > 1 site)	1 (2)
Venous thrombosis with swelling, ulceration, *or* venous stasis	1
Gastrointestinal	
Infarction or resection of bowel below duodenum, spleen, liver, or gall bladder ever, for any cause (score 2 if > 1 site)	1 (2)
Mesenteric insuffiency	1
Chronic peritonitis	1
Stricture *or* upper gastrointestinal tract surgery ever	1
Musculoskeletal	
Muscle atrophy or weakness	1
Deforming or erosive arthritis (including reducible deformities, excluding avascular necrosis)	1
Osteoporosis with fracture or vertebral collapse (excluding avascular necrosis)	1
Avascular necrosis (score 2 if > 1)	1 (2)
Osteomyelitis	1
Skin	
Scarring chronic alopecia	1
Extensive scarring or panniculum other than scalp and pulp space	1
Skin ulceration (excluding thrombosis) for >6 month	1
Premature gonadal failure	1
Diabetes (regardless of treatment)	1
Malignancy (exclude dysplasia) (score 2 if > 1 site)	1 (2)

Damage (nonreversible change, not related to active inflammation) occurring since onset of lupus, ascertained by clinical assessment and present for at least 6 month unless otherwise stated. Repeat episodes must occur at least 6 month apart to score 2. The same lesion cannot be scored twice
